# Unraveling the intricate dance of the Mediterranean diet and gut microbiota in autoimmune resilience

**DOI:** 10.3389/fnut.2024.1383040

**Published:** 2024-05-16

**Authors:** Christina Tsigalou, Avgi Tsolou, Elisavet Stavropoulou, Theocharis Konstantinidis, Efterpi Zafiriou, Efthymios Dardiotis, Alexandra Tsirogianni, Dimitrios Bogdanos

**Affiliations:** ^1^Laboratory of Hygiene and Environmental Protection, Medical School, Democritus University of Thrace, University Hospital, Alexandroupolis, Greece; ^2^Laboratory of Molecular Cell Biology, Cell Cycle and Proteomics, Department of Molecular Biology and Genetics, Democritus University of Thrace, Alexandroupolis, Greece; ^3^Department of Dermatology, Faculty of Medicine, School of Health Sciences, University of Thessaly, Larissa, Greece; ^4^Department of Neurology, Faculty of Medicine, School of Health Sciences, University of Thessaly, Larissa, Greece; ^5^Department of Immunology-Histocompatibility, Evangelismos General Hospital, Athens, Greece; ^6^Department of Rheumatology and Clinical Immunology, Faculty of Medicine, School of Health Sciences, University of Thessaly, Larissa, Greece

**Keywords:** autoimmune diseases, autoimmunity, dysbiosis, Mediterranean diet, microbiome

## Abstract

The nutritional habits regulate the gut microbiota and increase risk of an autoimmune disease. Western diet is rich in sugars, meat, and poly-unsaturated fatty acids, which lead to dysbiosis of intestinal microbiota, disruption of gut epithelial barrier and chronic mucosal inflammation. In contrast, the Mediterranean Diet (MedDiet) is abundant in ω3 fatty acids, fruits, and vegetables, possessing anti-inflammatory properties that contribute to the restoration of gut eubiosis. Numerous studies have extensively examined the impact of MedDiet and its components on both health and various disease states. Additionally, specific investigations have explored the correlation between MedDiet, microbiota, and the risk of autoimmune diseases. Furthermore, the MedDiet has been linked to a reduced risk of cardiovascular diseases, playing a pivotal role in lowering mortality rates among individuals with autoimmune diseases and comorbidities. The aim of the present review is to specifically highlight current knowledge regarding possible interactions of MedDiet with the patterns of intestinal microbiota focusing on autoimmunity and a blueprint through dietary modulations for the prevention and management of disease’s activity and progression.

## Introduction

There is a widely held belief that the Mediterranean Sea provides an ideal temperate environment, characterized by favorable conditions in temperature, humidity, and sunlight, for the countries and nations in its vicinity. The prevailing dietary habits among the inhabitants of the Mediterranean Sea basin are believed by scientists to contribute to a healthy way of life. From the early 1960s onwards, it became evident that individuals in the Mediterranean region adhered to a consistent eating pattern with subtle variations, commonly referred to as the “Mediterranean Diet” (MedDiet). However, it wasn’t until 1993 that the Harvard School of Public Health, Ordway’s Preservation and Exchange Trust and the European Office of World Health Organization, together with Greek researchers introduced the MedDiet Pyramid, based mainly on the eating patterns of Crete Island and Southern Italy (1, 2). The Mediterranean Diet (MedDiet) emphasizes a plant-based approach, with significant consumption of vegetables, cereals, nuts, and fruits, providing ample fiber. It includes smaller quantities of animal products, with a preference for seafood and fish.

Over the last 15 years, research has yielded a wealth of knowledge regarding human microbiome. The inconceivable diversity and abundance of all living organisms (microbes, fungi, viruses, parasites), which co-evolved with humans for thousands of years and inhabit the human body was revealed by next generation technologies. Observations in both experimental models and in humans struggle to define the precise relations and interactions between microbiota and health, as well as their correlation to different diseases. Symbionts and pathobionts are in a constant battle within gut microbiota contributing to a final state of symbiosis or dysbiosis. Different external and internal factors affect the composition of gut microbiome and diet appears to be one of the most important. Dietary habits have figurative results in intestinal microbiota and MedDiet seems to favor specific phyla while suppressing others (3).

It is widely-recognized that dietary shifts largely impact on microbial populations, which can, in turn, modulate innate and adaptive immunity (4). Modern techniques, as Next Generation Sequencing (NGS) managed to provide evidence for different stages of dysbiosis related to various immune-mediated and autoimmune diseases. However, our comprehension of the cause-and-effect model and the exact outcomes and implications of these interconnections remains limited (5, 6).

The literature research process involved searching the PubMed Database using Boolean operators (AND, OR, NOT) and combinations of keywords related to Autoimmune Disease, Autoimmunity, Dysbiosis, Mediterranean diet, Microbiome, and Intestinal Microbiota. Articles were included based on their relevance to how the Mediterranean diet influences intestinal microbiota patterns and their implications for autoimmunity. We refined our investigation to encompass studies published in English, exclusively concentrating on those conducted on human subjects. Our focus was specifically on articles discussing the Mediterranean diet as a whole, while excluding studies centered on specific foods within this dietary pattern.

The objective is to offer a comprehensive overview exploring the interplay between the Mediterranean Diet and Gut Microbiota in Autoimmune Resilience. The review aims to synthesize information in these areas, focusing on how the Mediterranean Diet influences intestinal microbiota patterns and their implications for autoimmunity. Additionally, it seeks to outline dietary modulations for preventing and managing autoimmune diseases.

Discussion covers the pathophysiology of autoimmune diseases, modulation of microbial dysbiosis, and attenuation of autoimmune-related inflammation. It’s evident that diet impacts various aspects of innate and adaptive immunity through diverse mechanisms, microbiota modulation to enhance the effectiveness of dietary interventions in managing autoimmune diseases. Articles were excluded if they lacked measurement methods and outcomes or focused exclusively on children and adolescents.

On a global scale, we seek to encapsulate the probable connections between adhering to the Mediterranean Diet (MedDiet) and the intestinal microbiota, particularly in the context of autoimmunity. Additionally, we explore potential dietary adjustments for the early prevention and effective management of autoimmune diseases.

## Special features of the Mediterranean diet and links to disease prevention or improvement

Mediterranean coastal countries have incorporated in their traditional food habits for years a healthy style of cooking and eating. As previously stated, the key components of MedDiet emphasize firstly, on the consumption of fruits and vegetables, nuts and whole grains, accompanied by healthy fats, mainly olive oil, spices and herbs, and secondly, on the small quantities of red meat and larger portions of fish and poultry. Additionally, the moderate drinking of red wine and physical activity/exercise is of great importance. In 2010, UNESCO introduced an expanded definition of the Mediterranean Diet (MedDiet), encompassing “a set of skills, knowledge, practices and traditions ranging from the landscape to the table, including the crops, harvesting, fishing, conservation, processing, preparation and, particularly, consumption of food” (7).

Large-scale projects demonstrated that the advantageous effects on the well-being of the participants seem to be attributed mainly to healthy mono-unsaturated fatty acids (MUFAs) in olive oil and flavonoids in red wine, nuts, spices and more (8–11). These effects are accomplished through the anti-inflammatory and anti-oxidative actions of these components, leading to the decrease of inflammation and oxidative stress (8). Yet, it’s important to also highlight the significant contribution of fiber from fruits and vegetables (1). Fiber plays a crucial role in digestive health and disease prevention (3). Overall, the Mediterranean Diet’s holistic approach, including a variety of nutrient-dense foods, has been linked to numerous health benefits, underscoring its importance for overall well-being and disease prevention.

Research conducted over the last two decades has consistently shown that adherence to MedDiet is beneficial for preventing or alleviating various inflammatory diseases. The lion’s share of the studies underlines the significant role of the Mediterranean dietary pattern in preventing cardiovascular diseases (9), type II Diabetes (10), obesity and metabolic syndrome (11, 12). Moreover, increased interest in the diet’s results on human morbidities underscores the effect of prolonged survival of the Elderly (13), better sleep and academic performance in teens (14), increased vitamin D levels, improvement of neck bone mineral density in adults who already had osteoporosis (15), longer length of telomeres in women (16), decreased depression in the Elderly (17), lower risk of fatty liver (18), lower risk of aggressive prostate cancer (19), prevention of colon cancer (20), reduced incidence of gestational diabetes and premature births (21), longevity (22), and improvement of arthritis symptoms (7). Studies suggest that the essence of the Mediterranean Diet’s benefits lies in the combined consumption of multiple health-promoting foods rather than strict adherence to a specific eating pattern. The protective effects of specific foods or nutrients in the diet appear to be less significant in combating diseases compared to embracing the entire dietary plan (23).

## Effects of diet on gut microbial communities

**Figure 1:**
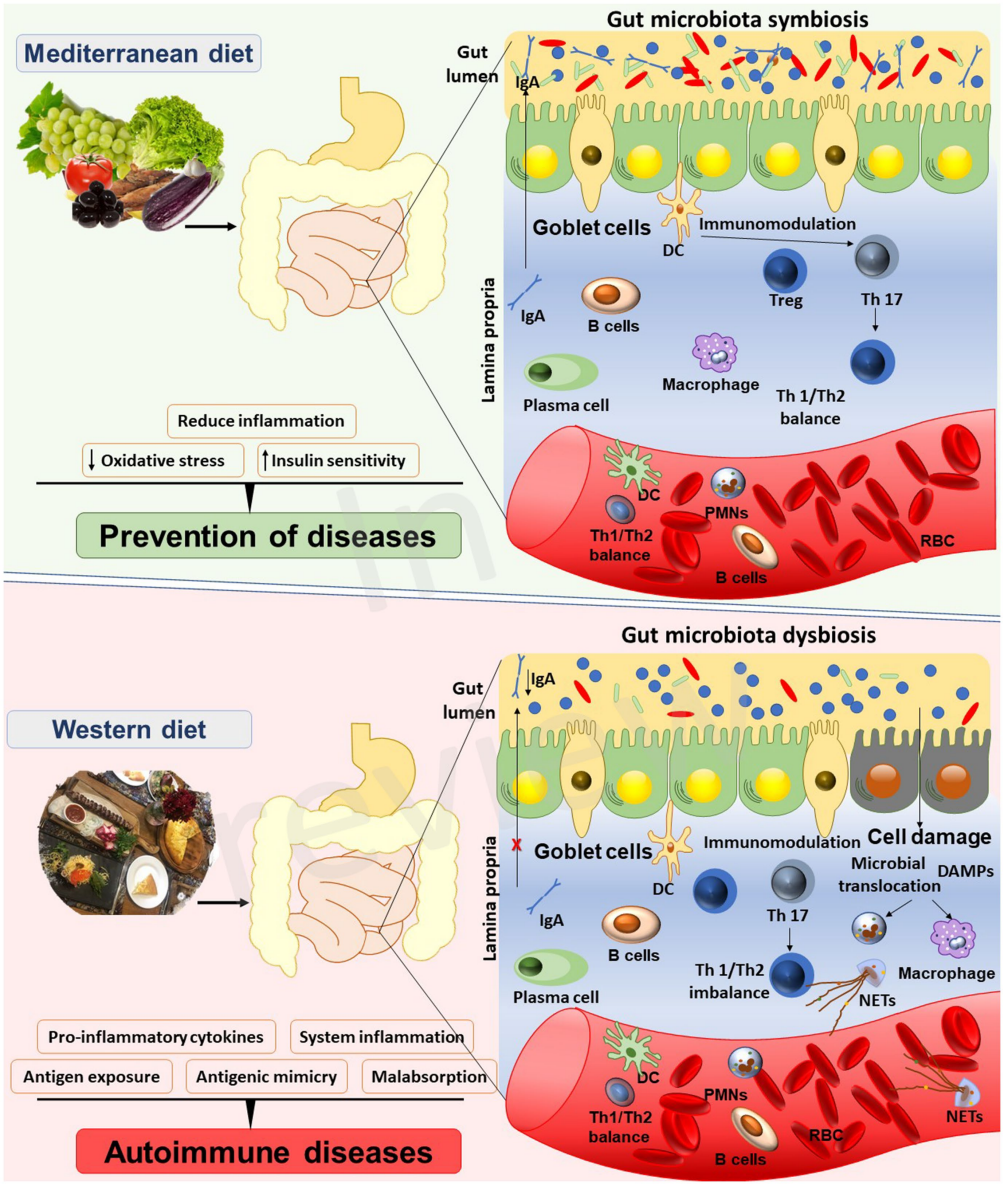
Interplay between the Mediterranean diet, gut microbiota, and autoimmune diseases.

According to Hippocrates, the father of modern medicine, ‘all diseases begin in the gut’. Centuries later, the truth of this wisdom has been unraveled through numerous studies, intricate reflections, and ambitious projects. Culture-independent techniques, as NGS in collaboration with bioinformatics, successfully accomplished the characterization of the inhabitants of our body from various anatomical sites concluding that the gut is, by far, the most populated site of the human body (24). Nowadays, it is well recognized that nutritional habits regulate the gut microbiota, and may through its effects on microbiome, have an impact on homeostasis and immune response and autoimmunity (Figure 1).

Advancements in technology and research have led to new insights, particularly in distinguishing between the microbiome and microbiota, two terms still under debate (25). Recently, there has been a resurgence in the original definitions proposed by Whipps et al. in 1988 (26). The microbiota, as defined, represents a characteristic microbial community within a well-defined habitat, characterized by specific physiochemical properties. It encompasses not only the microorganisms themselves but also their activities, forming distinct ecological niches. This dynamic and interactive micro-ecosystem undergoes temporal and spatial changes and is intricately linked with macro-ecosystems, including eukaryotic hosts, playing a crucial role in their functionality and health.

On the other hand, the microbiome consists of a collection of microorganisms from various kingdoms (Prokaryotes such as Bacteria and Archaea, as well as Eukaryotes like Protozoa, Fungi, and Algae). Its scope extends beyond the organisms themselves to include their activities, encompassing microbial structures, metabolites, mobile genetic elements (such as transposons, phages, and viruses), and relic DNA, all of which are influenced by the environmental conditions of the habitat.

Large-scale studies have dictated that different eating patterns promote a variety of gut microbiota composition and diversity and highlight diet as one of the most significant influencing factors (3, 6, 27, 28).

Different dietary practices, alterations in dietary components -such as fats, proteins and carbohydrates-, food complements as probiotics and prebiotics, even salt composition, can all modulate the gut microbiota (29). However, such changes can exert an effect not just at microbial species level but also at phyla differences (30).

The majority of study findings are based on animal experiments, particularly the reshaping of the gut microbiome due to a Western diet, resulting in a significant expansion of the Mollicute lineage within the Firmicutes, alongside a prevailing reduction in Bacteroidetes in the microbial community. Furthermore, research in both animals and humans indicates that increased fructose consumption contributes to small intestine bacterial overgrowth and elevated intestinal permeability, leading to high levels of endotoxins (31–35). Nevertheless, the supplementation of prebiotics has the potential to reverse microbial population shifts induced by a high-fat diet in obese mice (36). Furthermore, mice with an elevated protein intake exhibited notable increases of *Lactobacillaceae/Lactobacillus* and decreased *Clostridiaceae/Clostridium* within their gut microbiota, with the extent of these changes being dose-dependent (37). In humans, gut microbiota varies at the species level, depending on fat and fiber or carbohydrate consumption, between omnivores and vegetarians, or even among vegetarian subgroups (3). On the whole, dietary patterns play a role in shaping the diversity of gut microbiota. This diversity lies on higher taxonomy levels, as phylum, family, and genus, rather than at the species level. Changes in individual dietary components, such as gluten, may also change composition and function of the gut microbiome and host physiology of healthy individuals (38). Investigating geographical and socio-economic differences in various regions, numerous studies shed light on how diet influences gut microbiota, comparing a spectrum of dietary habits (39–41).

Interestingly, a large-cohort study on US immigrants from non- Western countries led to a relatively fast and enduring loss of gut microbiome diversity, accompanied by the decline of bacterial enzymes related to plant fiber degradation and the displacement of native strains and functions within the first 9 months of immigration and, as a result, predisposition of individuals to obesity and metabolic diseases. Substantial disruptions in the gut microbiome can be partially explained by dietary variations (42).

## Gut microbiome’s link to autoimmunity

It seems that apart from the identification of all taxa of microbes, the ultimate challenge is to define the benefits and drawbacks of the ‘healthy gut’. The human microbiota, and the gut in particular, has been deemed an “essential organ,” containing approximately 1,000 different microbial species, and counting for over 150 times more genes than those of the whole human genome (43, 44).

The pivotal role of human microbiota in health and disease involves a diverse array of functions. The microbiota assists energy extraction from food, supplies unique enzymes and biochemical pathways to the human body, and acts as a physical barrier against foreign pathogens through antimicrobial substances or space competition (45–47). Last but not least, the gut microbiota is essential for the normal development of both the intestinal mucosa and the host humoral and cellular immune system, as signals and metabolites from the microorganisms are sensed by cells of the innate immune system and are recognized as physiological responses (48–50). The term “autoimmunity” refers to activation of adaptive immune responses, with the involvement of T and B lymphocytes against self-antigens, i.e., against its own healthy cells and tissues. The actual causes of autoimmunity are not fully understood, but various environmental factors, including diet, lifestyle and infections, along with the genetic background of the host, appear to play a crucial role (51, 52). The initiation of adaptive immune responses is rooted in the connection between the innate and adaptive immune systems. It is the innate immune system that can discriminate between self and non-self-antigens, whereas the adaptive immune system recognizes either native antigens or peptides presented in the context of major histocompatibility complex molecule (MHC) (53). Therefore, when a microbial pathogen or parasite invades the host, both the adaptive immune system and the innate system respond accordingly. Studies in both humans and animal models designate the involvement of commensal microbiota in autoimmunity (54, 55).

The impact of the microbiota on autoimmune diseases, which rely on the innate-adaptive immune system connection, can range from being neutral to being essential for the initiation of autoimmunity. The loss of immune tolerance to self-antigens can occur due to changes in microbial composition. Consequently, the human microbiota becomes a crucial participant in the initiation and perpetuation of autoreactive immune responses, ultimately resulting in self-tissue destruction and the overt of autoimmune diseases (54, 55). Several factors contribute to the loss of immune tolerance and induction of autoimmunity by microorganisms, including molecular mimicry, bystander activation, and viral persistence with or without epitope spreading (56–60) focused, dominant epitope-specific immune response. This expansion targets self or foreign proteins, encompassing subdominant and/or cryptic epitopes on the same protein (intramolecular spreading) or other proteins (intermolecular spreading) (61).

Self-antigens can be a result of slightly changed antigens, even at an amino acid residue. As a result, the immune reaction will affect both the “wild” protein or the altered one (61, 62).

Microorganisms could trigger autoimmune responses through the expression of heat- shock proteins in their cells when under stress, such as high temperatures. These proteins can subsequently become targets of the immune system’s response. Heat shock proteins produced by microbes have the potential to trigger self-reactivity toward the host’s own heat shock proteins, potentially leading to autoimmune diseases. This self-reactivity toward heat shock proteins serves to protect the host against disease by regulating the induction and release of pro-inflammatory cytokines. Nevertheless, antibodies targeting self heat shock proteins have been implicated in the pathogenesis of autoimmune diseases such as arthritis and atherosclerosis (63).

In ‘*molecular mimicry’*, a shared immunologic epitope between a microbe and the host is the prerequisite for the initiation of cross-reactive immune responses. A notable illustration of molecular mimicry as a mechanism leading to the onset of autoimmune disease is evident in individuals with rheumatic fever after being infected with group A beta-hemolytic Streptococci. Analysis of infected hosts’ sera demonstrated the presence of antibodies reactive with heart, joints, brain, and skin. Moreover, patients’ antibodies are found to cross-react with streptococcal antigens, like the group A carbohydrate antigen, the M protein (a *Streptococcus*- related virulence factor) and to cross-react with myosin. Cross-reactive peptides from M protein and cardiac myosin may provoke the onset of autoimmune disease in mice with rheumatic heart disease (62). The mechanism of molecular mimicry can also operate at the T-cell level involving antigenic epitopes of human and foreign origin which serve as targets of CD4 and CD8 T-cell responses. Previous studies of our group have meticulously addressed the role of molecular mimicry in the induction of autoimmune diseases, primarily affecting the liver and the gastrointestinal tract (62–82).

*‘Bystander activation/killing’* is another mechanism resulting in autoimmune diseases. Viral infections can activate Antigen-Presenting Cells (APCs) which, in turn, activate primed autoreactive T cells for the onset of autoimmune disease. Furthermore, initiation of bystander activation can be a result of virus-specific T cells. Bystander effect may lead to killing of the uninfected neighboring cells, that increases the immunopathology at the infected area (82). Finally, persistent viral infections may cause immunopathology, as a result of the permanent presence of viral antigens challenging the immune system (83).

## Adherence in MedDiet and its effects on microbiome related to autoimmune diseases: the case of rheumatoid arthritis

The Mediterranean diet is rich in fiber, antioxidants and vitamins and encompasses anti- inflammatory properties (84). Various data have demonstrated that consumption of cereals, fruits and vegetables, nuts and legumes, omega-3 polyunsaturated fatty acids in olive oil and moderate consumption of red wine flavonoids leads to the reduction of pro-inflammatory cytokines, the increase of anti-inflammatory cytokines and the decrease of oxidative stress (83, 84). It has been shown, that adherence to a Mediterranean pattern diet leads to the reduction of CRP and TNF-α levels (85, 86).

Several studies investigated the role of MedDiet in Rheumatoid Arthritis (RA), a common autoimmune rheumatic disease. Highlighting the immunopathological characteristics of rheumatoid arthritis (RA), underscore the distinctive cytokine profiles associated with the condition, notably the heightened levels of Th1/Th17 cytokines and the compromised function of Tregs (87). Immune dysregulation, and imbalance between the function, differentiation, and regulation of Th17 and Treg cell plays a highlighting role in disease onset (87) disorders, rheumatoid arthritis is associated with oxidative stress. This refers to a state being a quintessential example of chronic inflammatory autoimmune where the level of reactive oxygen species gradually rises, either due to increased production, diminished antioxidant defenses, or both, ultimately leading to disruptions in redox signaling (88). Data suggest that adopting a Mediterranean Diet model reduces the inflammatory activity of the disease, enhances functionality, and improves the quality of life for patients with RA (89). Another study claims that adherence to MedDiet is associated with decreased disease activity, improved physical function, and heightened vitality individuals with RA (87). Subsequent studies have yielded inconsistent findings (90–98). A recent systematic review analyzing the data so far provided from prospective human studies, concluded that MedDiet has beneficial effects in reducing pain and increasing physical function in people with RA but underline that there is insufficient evidence to support the widespread recommendation of the Mediterranean Diet for the prevention and management of RA (7).

Data from studies on short term MedDiet and fasting revealed no significant impact of these diets on the gut microbial profile in individuals with RA or fibromyalgia (98).

Recent studies have underscored the importance of long-term adherence, lasting more than 3 months to MedDiet is required to produce significant diversity in the gut microbiome of overweight omnivores (99). The CARDIVEG study reported that MedDiet significantly alters the abundance of *Lachnoclostridium, Enterorhabdus* and *Parabacteroides*, while vegetarian diet significantly disturbs the abundance of *Streptococcus*, *Anaerostipes*, *Clostridium sensustricto*, and *Odoribacter* (99).

A recent study suggests the potential role of *P. copri* in the preclinical evolution and pathogenesis of synovitis in RA, based on the analysis of anti-*P. copri* antibody responses in different RA cohorts (100).

RA drugs include disease-modifying antirheumatic drugs (DMARDs), target cytokines and immune responses. Patients usually initiate treatment with conventional DMARDs such as methotrexate. However, exploring alternative approaches like probiotics, prebiotics, herbal remedies, and dietary interventions is crucial for discovering new avenues for treating the disease.

Modulating gut microbiota holds promise as a potential approach for treating rheumatoid arthritis (RA) microbiota transplantation (FMT) is being investigated for its ability to rebalance the gut microbiota and improve RA symptoms (101).

In rheumatoid arthritis (RA), the presence of *Prevotella* spp., particularly *P. copri*, exacerbates the disease by promoting proinflammatory metabolite production. Colonization with *P. copri* and a fiber-rich diet leads to dysbiosis, characterized by metabolites like succinate and fumarate, worsens RA symptoms (102). Conversely, a Mediterranean diet’s effectiveness in RA treatment may vary based on gut microbiome personalized treatment approaches and may aid in identifying predictive biomarkers microbiota in mediating dietary effects on RA could help elucidate why certain dietary enhancing the efficacy of dietary interventions in RA management, ameliorate and exacerbate colitis in animal models. While they have the potential to improve colitis symptoms, caution is advised as certain prebiotic fibers may contribute to gut dysbiosis and lead to excessive production of colonic butyrate, potentially worsening inflammatory bowel disease (IBD) (103). The fermentation products generated by consuming high-fiber diets (and possibly Mediterranean diets) can have adverse effects, particularly in the presence of intestinal inflammation. For instance, the production of butyrate after diet fermentation may exacerbate inflammation by promoting NLRP3 activation.

### Meddiet lead to changes of intestinal short-chain fatty acids (SCFA)

Such studies on gut microbiome changes in patients with RA adherent to MedDiet have not been performed so far, but several data from research conducted on the gut microbiome and its relation to RA may be relevant. Several studies have assessed changes of the microbiota in relation to MedDiet in patients with RA. An early study suggested that changes of intestinal Short-Chain Fatty Acids (SCFA) from the microbiota are not necessarily correlated with clinical improvements and disease activity in RA (104).

An association between MD and increased SCFAs production is well known (105). Rich fiber foods like fruits, vegetables, and legumes, commonly consumed by those following the Mediterranean Diet, are broken down by Firmicutes and Bacteroidetes bacteria. This process results in the production of elevated levels of fecal Short-Chain Fatty Acids (SCFA) (106, 107).

Among these SCFAs, butyrate, extensively studied for its functional role, is produced during the fermentation of dietary fiber by the anaerobic intestinal microbiota. Butyrate has beneficial effects on intestinal barrier integrity, as it enhances the expression of tight junction proteins. Moreover, it helps prevent deleterious intestinal permeability and bacterial translocation, thereby exerting a protective influence on the initiation of pro- inflammatory responses (106, 108, 109). To relevance in collagen-induced arthritis, an animal model of RA, butyrate is able to suppress RA features and this is achieved via a butyrate- mediated increase of IL-10 producing Tregs and a decrease of Th17 (110). However, not all Mediterranean type of diets increase SCFAs, and butyrate in particular at the same extent, and this may have an impact in their ability to suppress anti- inflammatory immune responses or to influence intestinal microbiota-related influence of the immune system.

Modified Mediterranean type enriched for SCFA production are increasingly popular but their effect in RA has not been assessed. Omnivores who consume a MedDiet-pattern diet rich in fruit, legumes and vegetables not only have increased SCFAs (106), but also decreased trimethylamine N-oxide (TMAO), a microbial metabolite the precursors of which are carnitine and choline which are primarily found in foods of animal origin (106, 111).

Several microbial genera, like L-*Ruminococcus*, have been linked to the intake of animal proteins such as a diet plenty in red meat consumption and increased TMAO levels. This is very interesting, in view of recent data demonstrating a twofold to threefold increased abundance of *Ruminococcus gnavus* in patients with spondylarthritis and to a lesser extent in RA patients compared to healthy controls (112).

However, the most notable association over the last few years is that linking *P. copri* the TMAO-producing anaerobic, Gram-negative Bacteriodetes, with the development of RA, an association thoroughly reviewed elsewhere (113, 114). *Prevotella* spp. are abundant in the periodontium, the intestine, and the respiratory system and its heightened presence is deemed a risk factor for RA and features associated with RA, such as cardiovascular risk- events (113, 115–118). Furthermore, it may impact the metabolism of the microbiota to reduce the effectiveness of the common disease-modifying anti-rheumatic drug (DMARD) methotrexate (119). The question arises as to whether adherence to MedDiet alone or in combination with other diet supplementation can alter gut dysbiosis to a state that *Prevotella* spp. are not dominant (62, 120, 121). This event would stop the vicious circle of immunological events that take place, *Prevotella* being in the center of it and could prevent from RA.

A recent elegant study has shown that the microbiota of individuals in pre-clinical early RA stages had significantly altered fecal microbiota composition compared with their first- degree relatives (FDRs) (116). In these pre-clinical RA individuals, who had either developed anticitrullinated peptide antibodies or rheumatoid factor positivity, and/or exhibited symptomatology and features associated with possible RA in the ‘pre-clinical stages,’ their feces were significantly enriched in the bacterial family *Prevotellaceae*, particularly *Prevotella* spp., compared to their first-degree relatives (FDRs) (116). These data clearly demonstrate that *Prevotella* spp. enrichment in early RA and very early RA may indeed be a characteristic feature of these subclinical phenotype raising the expectation that *Prevotella* spp. are pathogenically relevant to the development of the disease, from early stages, rather than consequence of established disease state.

We recently reviewed the existent literature tackling this topic and thoroughly discussed mechanistic scenarios, which could implicate *Prevotella* species in the establishment of RA (122). We underlined the decisive role of *Prevotella species* in the potential induction of either ACPA-positive or ACPA-negative RA. This contrasts that of the well-known association of *P. gingivalis* and ACPA-positive RA and the inability of this oral commensal to explain the induction of ACPA-negative RA. Though several components of the *Prevotella*-host interactions are still puzzling and improperly explored, some of the features linking *P. copri*, as well as oral *Prevotella* species, to RA are striking. One of the most fascinating has recently been obtained by Pianta et al. (123). These authors using liquid chromatography–tandem mass spectrometry identified two novel RA autoantigens, targeted by half of the ACPA-pos and ACPA-negative patients with RA. The two autoantigens were the N-acetyloglucosamine- 6- sulfatase (GNS) and filamin A (FLNA). Of interest, both GNS and FLNA were expressed in synovia, a finding that supports the notion that they could be relevant to immune-mediated tissue destruction. The former also appeared to be citrullinated, which makes it likely target of ACPA antibodies. Data indicating that antibody concentrations of these autoantibodies are correlated further supports the likely association of anti-GNS antibodies with ACPA (123). These autoantigens were recognized by more than half of the RA patients, and were also present in ACPA-negative RA. Of immunological relevance, Pianta et al. (123) found that the epitopic regions of GNS and FLNA not only are highly homologous to *Prevotella copri* but are also targeted by B and T-cells responses, also cross-recognizing the *Prevotella* homologs. Thus, there is evidence that a molecular mimicry mechanism is in operation, which could account for the induction of those autoantibodies (123). Of relevance, molecular mimicry involving an oral *Prevotella* sp. and collagen I, have been previously reported and has been considered a likely trigger for chronic periodontitis and possibly inflammatory (124).

The GNS peptidyl sequence was highly homologous to a sequence from sulfatase proteins of the *Prevotella* sp. and *Parabacteroides* sp. Finding marked homologies between human and microbial highly conserved proteins is extremely common and the homology reported by Pianta et al. belongs to this category (123). In a similar vein, homologies between human and microbial heat shock proteins and human and microbial 2-oxo-acid dehydrogenase complexes have been identified and suggested as triggers of various organ and non-organ specific immune-mediated and autoimmune diseases. Because they are extremely common, several investigators, including authorities in the field, suggested that molecular mimicry involving such homologs must not be regarded as a perpetuator of autoimmunity.

Similarities between human GNS and *Prevotella* were shown by Pianta et al. (123). The same human GNS sequence had also marked amino acid similarity with the gut commensal *Parabacteroides* sp. Using a BLAST program, they investigated for additional similarities that could be potentially homologous to the human GNS epitope. Microbial mimics that have similarities to the core epitope region of the human GNS epitope were also presented.

Particularly, the pentameric -FFMMI- 224-228 aa of human GNS is contained in the transmembrane protein of *Streptococcus gordonii* (aa 43–47), transcriptional regulator of *Lactobacillus casei* (aa 464-468), acetyl-CoA carboxylase, carboxyl transferase subunit beta of *Clostridium cellulolyticum* H10 (aa 133-137), hypothetical protein of *Vibrio* phage KVP40 (aa 23-27) and several other foreign proteins, suggesting that many other triggering factors may really exacerbate GNS-specific autoreactivity in RA by molecular mimicry. Thus, Pianta’s homologs (123) cannot be disregarded as the relevant mimics are targets of cross-reactive responses and the humoral responses against the microbial peptides are correlated with disease-specific autoantibodies, as ACPAs. Moreover, gut dysbiosis leads to immunologic alterations, which are pivotal for the loss of immunological tolerance to RA-specific autoantigens. This was achieved in a stepwise manner and that several immunological mechanisms are involved, molecular mimicry being just one of those. Firstly, an external parameter leads to changes in the gut microbiome, as well as changes on the microbiome of the oral cavity and an establishment of gut dysbiosis (123). This parameter or combination of parameters could be drugs, infections, changes of diet habits from Mediterranean diet to Western diet and more. Gut dysbiosis, in turn, leads to the establishment of an immunological environment, which alter the composition of regulatory T and B cells and diminishes their capacity to suppress autoreactive immune responses and augments pro- inflammatory Th17 responses. The enrichment of specific species, such as *Prevotella*, has additional consequences, notably the activation of the immune system against a gut microbe. This can initiate anti-*Prevotella* responses, which, through mechanisms like molecular mimicry and others, in conjunction with various factors, may lead to the induction of autoreactive responses. This initiation can lead to the development of autoimmune disease only in susceptible individuals (125).

Of indirect relevance to the topic, among the American indigenous populations, the Canadian Inuit population has the lowest age-adjusted prevalence of RA (at 0.65%, with an incidence of 48.2 per 100,000 per year) (126). This is of interest because a microbiome study has found that *Prevotella* spp., were enriched among the Inuit consuming a Western diet.

However, the gut microbiome of Inuit consuming a traditional high-fiber diet (127) had significantly less genetic diversity within the *Prevotella* genus, compared to the Inuit consuming a Western diet further, indicating that a low-fiber diet might not only select against *Prevotella* but also decrease its diversity, a factor which could be relevant to the induction of autoreactive responses implicating *Prevotella* in inflammatory arthritis.

A recent study has shown that berberine modulates gut microflora and exerts an anti- inflammatory effect on collagen-induced arthritis (128). This is achieved because the abundance of *Prevotella* is diminished and the abundance of butyrate-producing bacteria in CIA rats is increased (128).

Nonetheless, a study examining changes in gut microbiota associated with the MedDiet discovered a greater presence of Bacteroidetes and a lower Firmicutes–Bacteroidetes ratio in those with a higher Mediterranean Diet score (129). However, the study did not report significant differences in levels of *Bacteroides* and *Prevotella*, genera included in the Bacteroidetes phylum (129). Again, it is not clear whether long-term adherence to MedDiet may indeed exert an influence on the levels of *Prevotella,* which could in turn utilize beneficial effects in preventing from RA or ameliorating disease’s clinical features. Another study conducted an integrative analysis of distal gut microbiota composition and functions, as well as intestinal metabolites in Egyptian teenagers consuming Mediterranean Diet- related products. The study compared this cohort with a group of USA teenagers consuming a Western-type diet enriched in animal proteins, fats, and processed carbohydrates (130). The *Prevotella* enterotype predominated in gut microbial communities of the Egyptian teenagers while the *Bacteroides* enterotype dominated the USA cohort. As it was expected the intestinal environment of Egyptian teenagers was characterized by higher levels of SCFA, an increased prevalence of microbial polysaccharide degradation-encoding genes, and a higher proportion of several polysaccharide-degrading genera while the gut environment of the American children was enriched in proteolytic microbes and end products of protein and fat metabolism (130).

## Adherence in MedDiet and its effects on microbiome related to autoimmune diseases: the case of autoimmune disease of the central neuros system (CNS)

Multiple sclerosis (MS) and Neuromyelitis optica (NMO) is an autoimmune disease of the CNS, with some overlapping symptoms. A few studies have shown that both genetic and environmental factors may affect the etiology of the MS, including dietary habits. Moreover, the onset of the disease post microbial infections via molecular mimicry and bystander activation mechanisms is also a scenario (131, 132). The multifaced roles of gut microbiome on MS pathogenesis has been shown in a reciprocal way; germ free or gnotobiotic mice demonstrated considerably reduced susceptibility to experimental autoimmune encephalomyelitis (EAE), a MS model, while CNS disease may affect gut homeostasis. The gut-brain connection is largely supported by evidence on individuals with MS (133, 134).

Th17 cells are normally required for host defense against invaders, but they may have vicious effects in terms of autoimmunity, also suggested by their increased numbers in MS. The role of gut microbiota in Th17 cells and their IL-17 product, and their increased numbers of the latter ones in MS have been reviewed in detail (135, 136). On top of that, the main role of Tregs is the suppression of autoreactive T cells, thus maintaining peripheral tolerance. Although abundant, demonstrate reduced function in MS patients. Taken together, along with the outcome of several studies regarding gut microbiota composition in CNS patients, the gut of MS individuals is characterized by microbial dysbiosis, i.e. impaired intestinal microbiota (131). It is widely accepted that dietary habits affect the composition of human microbiota. High-fiber foods enhance gut populations of the Firmicutes and Bacteroidetes phyla, which produce short-chain fatty acids (SCFAs), which, in turn, suppress inflammation via Treg induction (137).

In 2016, the findings of a small, randomized control trial (RCT) of a Mediterranean-style dietary intervention for MS were published (138). Thirty-three Relapsing–Remitting MS (RRMS) patients were randomized into three groups: group 1 undertook a dietary intervention with vitamin D supplementation, group 2 started vitamin D without a dietary intervention, and group 3 underwent a dietary intervention with vitamin D and other nutritional supplements, including a multivitamin and fish oil supplement (138). The study failed to find a significant effect of the intervention on the Expanded Disability Status Score (EDSS) or the Fatigue Severity Scale (FSS) (138). Published data evaluating adherence to the Mediterranean Diet pattern, assessed using alternate MedDiet score, and the risk of an initial clinical diagnosis of the precursor of multiple sclerosis (MS), specifically central nervous demyelination (139). The researchers found that a Mediterranean diet, included unprocessed red meat, was associated with a reduced risk of demyelination in the Australian adult population. Their conclusion suggested that integrating unprocessed red meat into a Mediterranean diet might bring about beneficial effects for individuals at a high risk of multiple sclerosis (MS).

### Adherence in MedDiet and its effects on microbiome related to autoimmune diseases: the case of systemic lupus erythematosus (SLE)

Apart from MS and RA, there are other autoimmune diseases related with the gut microbiota, as Systemic Lupus Erythematosus (SLE), and the Inflammatory Bowel Disease (140–142). Concerning Systemic Lupus Erythematosus, there are conflicting results from a limited number of studies indicating a smaller Firmicutes-to-Bacteroidetes (F:B) ratio compared to healthy individuals. It appears that this ratio is not well-established as a clear cause or consequence, as it could be both simultaneously (143). Howbeit, *Enterococcus gallinarum*, member of gut commensals of the Firmicutes, appears to promote a lupus-like disease (140) In Inflammatory Bowel Disease (IBD), specifically in ulcerative colitis (UC) patients, *Faecalibacterium prausnitzii* is reduced in feces. In Crohn’s disease biopsies, *Faecalibacterium prausnitzii (F. prausnitzii)* is also detected (143).

In conclusion, upcoming research focusing on the influence of specific components or the entirety of the Mediterranean diet in ameliorating microbial dysbiosis and mitigating autoimmune-related inflammatory reactions holds significant importance. The diet seems to influence components of both innate and adaptive immunity through a myriad of mechanisms, either independently or collaboratively.

### Restoring microbiota through ‘nutraceuticals’

Nutraceuticals, a term coined by their “Godfather” Dr. Stephen De Felice in 1989, embody a fusion of nutritional and pharmaceutical concepts. They encompass products that are isolated or purified from foods. Established nutraceuticals include probiotics, prebiotics, omega-3 and -6 fatty acids, and others like polyphenols, phytoestrogens, flavonoids and antioxidants, with already recognized favorable effects under specific conditions (143–148). Nutraceuticals are “related” to the human microbiota that includes 6 taxonomic bacterial phyla with Firmicutes and Bacteriodetes occupying the 90% of the host’s colonized areas (149). Nutraceuticals include probiotics, which WHO defines as “live micro-organisms” which, when administered in adequate amounts, confer a health benefit on the host.

Probiotics produce short chain fatty acids (SCFAs), which are able to restore both population numbers and diversity of microbiota. *Lactobacillus* species may decrease or even prevent the symptoms of antibiotic associated diarrhea (AAD) (150), while a meta-analysis study showed positive effect of probiotics on AAD (151). Furthermore, *Lactobacillus, Bifidobacterium* or *Escherichia coli* species have positive impact on host against metabolic diseases or gastrointestinal disorders (149).

Prebiotics, on the other hand, are “dietary carbohydrates stimulating the development of gut bacteria or probiotics post external administration, having advantageous results on the host” (152). Breakdown of carbohydrates supply the body with SCFAs, − acetate, propionate and butyrate- which have a beneficial role on the composition and diversity of human microbiota (153). Short-chain fatty acids (SCFAs) have immunomodulatory effects, but the underlying mechanisms are not well understood. SCFAs promote the differentiation of Tregs and protect the integrity of the gut barrier function (153). Also, these fatty acids may regulate immune procedures through G-protein coupled receptors (GPCRs). Gut homeostasis is critically correlated to these specific metabolites as acetate, butyrate and propionate and its disruption could lead to Inflammation Bowel Disease (IBD). SCFAs are very promising interventions for IBD therapy as they reinforce the gut barrier (153). Another important epigenetic factor is the family of histone deacetylase (HDACs) and particularly ADACs inhibitors coming from diet components for example chrysin found in fruits, vegetables, olive oil and red wine (154, 155). Experimental studies present models with HDAC inhibitors for treating T-cell mediated autoimmune diseases (156). Yet, in IBD again particular diet compounds from MedDiet may regulate epigenetic changes to diminish inflammation and the cancer risk (157).

Moreover, the advantageous impact of prebiotics on host health comprises alterations in gut microbiota composition, immune host capacity, energy production, enhanced mineral absorption and better functions of the intestinal barrier (158). Administration of inulin or Fructo-oligosaccharides (FOS) on cancer patients demonstrated a positive impact of these components on gut populations of *Lactobacillus* and *Bifidobacteria* (159). Ingredients in onions and garlic may have beneficial effect on particular gut microbial species populations, and, on the other hand, lethal effects on pathogens like *E. coli* and *S. aureus* (160).

Phytoestrogens include flavonoids, which regulate the intestinal barrier and own antimicrobial effect against pathogens, being characterized as alternative antibiotics (161). Anthocyanins and flavonoids, found in fruits like grapes and apples, may prevent a wide range of diseases (162). Polyphenols, as quercetin, found in apples, grapes, onions, tomatoes, nuts and seeds, alters the gut microbiota in overweight mice fed with high-fat sucrose diet (163). Resveratrol, another polyphenol taken as a food supplement regulates gut microbiota dysbiosis caused by high-fat diet, by enhancing growth of *Lactobacillus* and *Bifidobacterium*, raising the ratio of Bacteroidetes/Firmicutes and by hampering growth of *E. faecalis* (164). Carvacrol and thymol, phenols in the aromatic plant *Oregano vulgare*, have antibacterial properties and ability to affect the gut microbiota and the immune status in animal models (165–167). Use of omega-3 and -6 polyunsaturated fatty acids, as supplements, alter gut microbiota composition, by increasing Bacteroidetes/Firmicutes ratio, restrains growth of pathogenic bacteria like *Helicobacter, Firmicutes, Pseudomonas* sp., thus, avoiding immunological disturbances (165).

## Conclusion

Taking together, there appears to be a strong correlation between dietary habits, whether in the form of foods or dietary supplements like nutraceuticals, the modulation of gut microbiota, and the critical role of human commensals in disease prevention and regulation. This extends to the onset and development of various immunological disturbances, including autoimmune and metabolic diseases. The Mediterranean diet is based on fruits, vegetables, seeds, nuts, fish, whose composition is rich in prebiotics, phytoestrogens as flavonoids, polyphenols and omega −3 and − 6 polyunsaturated fats. Several studies have established a correlation between Mediterranean dietary habits and favorable effects on gut microbiota composition. This association suggests a potential positive impact on the risk and progression of inflammatory diseases. Consequently, it could be critically important to advocate for the adoption or reinforcement of Mediterranean dietary habits. Such habits may contribute to the modulation of microbial homeostasis, mitigate the effects of pathogen invasions, and influence the inflammatory pathway, ultimately benefiting health. However, further studies, on its individual components and/or total/overall compounds of the Mediterranean diet “philosophy” on health’s benefit via microbiota homeostasis should be conducted, to address the effect of the diet and its constituents in the prevention and clinical management of patients affect with specific autoimmune disorders.

## Author contributions

CT: Conceptualization, Data curation, Writing – original draft. AvT: Data curation, Resources, Writing – original draft. ES: Writing – review & editing. TK: Data curation, Resources, Software, Writing – review & editing. EZ: Investigation, Resources, Software, Writing – review & editing. ED: Formal analysis, Investigation, Project administration, Writing – review & editing. AlT: Formal analysis, Methodology, Validation, Writing – review & editing. DB: Supervision, Validation, Writing – original draft.
